# Changes in Chemical Composition and Accumulation of Cryoprotectants as the Adaptation of Anholocyclic Aphid *Cinara tujafilina* to Overwintering

**DOI:** 10.3390/ijms22020511

**Published:** 2021-01-06

**Authors:** Roma Durak, Joanna Depciuch, Ireneusz Kapusta, Joanna Kisała, Tomasz Durak

**Affiliations:** 1 Department of Experimental Biology and Chemistry, University of Rzeszów, Pigonia 1, 35-310 Rzeszow, Poland; jkisala@ur.edu.pl; 2 Institute of Nuclear Physics Polish Academy of Science, 31-342 Krakow, Poland; joanna.depciuch@ifj.edu.pl; 3 Institute of Food Technology and Human Nutrition, University of Rzeszów, Zelwerowicza 4, 35-601 Rzeszow, Poland; ikapusta@ur.edu.pl; 4 Laboratory of Plant Physiology and Ecology, University of Rzeszów, Rejtana 16c, 35-959 Rzeszow, Poland; tdurak@univ.rzeszow.pl

**Keywords:** aphid, cryoprotectant, insect, overwintering, sugars, polyols

## Abstract

One of the consequences of climate change is the expansion of insects’ ranges. Colonization of new habitats forces insects to adapt to new conditions, such as low temperatures in winter. *Cinara tujafilina* is a thermophilic anholocyclic aphid species, which reproduce exclusively parthenogenetic throughout the year, including winter. On the areas where the populations of *C. tujafilina* had expanded, it demonstrated its adaptation for surviving colder winters. Based on analyses of changes in body chemical composition using Fourier transform infrared (FTIR) and changes in cryoprotectant content using high performance liquid chromatography (HPLC), we showed how aphid *C. tujafilina* adapted to overwintering as an active stage. In the FTIR spectrum of the winter type of *C. tujafilina*, higher peak values originating from the carbohydrates, proteins and lipids, were observed. Glucose, trehalose, mannitol, myo-inositol and glycerol were identified in the aphid body in winter as main putative cryoprotectants to increase the insects’ tolerance to cold. The complex sugar-polyol cryoprotectant system facilitates aphids’ survival in unfavorable low temperatures.

## 1. Introduction

Climate changes affect the distribution and life cycles of insects. These changes can also affect the migration periods, phenology of species, number of generations per year, period of colonization of the host plants and fertility. One of the consequences of climate change is also the expansion of insect ranges [1,2,3]. The spread of species and colonization of new habitats forces insects to adapt to new conditions, such as low temperatures in winter. Insects have developed behavioral and physiological adaptations to survive low temperatures during winter months. One of the most common adaptations is cold hardiness, the ability to endure temperatures below 0 °C. One of the main biochemical mechanisms that allows insects’ to survive the uncomfortable low temperatures is synthesis of low molecular weight sugars, polyols (sugar alcohols) and some amino-acids, as cryoprotectants [4,5]. Amino-acids and sugars (especially trehalose) have cryoprotecting properties, by protecting enzymes and membrane structure under stress caused by low temperatures [5]. High concentrations of polyols not only decrease the temperature of crystallization of an insect’s body fluids but also stabilize the state of proteins at low temperatures, even when accumulated in relatively low concentrations [6,7]. Polyols regulate the amount of water available for freezing and, reduce the extent of cell dehydration caused by extracellular freezing. They protect the structures of biological membranes and proteins during dehydration caused by freezing [4,7]. Trehalose, glycerol and myo-inositol have been shown to be the main cryoprotectants in insects [6,7]. Many authors point also to an increase in sugars and polyols during insect diapause [6].

Aphids, like other insects, have life strategies to avoid low temperatures during winter [1]. Most female aphids (holocyclic) produce eggs in late autumn, which then overwinter. Another life-cycle strategy is the anholocyclic life cycle, which entails exclusively parthenogenetic reproduction throughout the year, including winter [8]. Most of the population or species of anholocyclic aphids die in the autumn when ambient temperatures drop significantly. Anholocyclic species have to overwinter on host plants as active morphs. Aphid eggs are more resistant to low winter temperatures than other developmental stages. The eggs of some species can survive at temperatures below −30 °C [9]. This is the reason why the holocyclic aphid species inhabit the areas with severe winters, whereas anholocyclic species more often occur in areas with milder winters or may overwinter inside glasshouses [10,11].

As a result of climate warming, anholocyclic species may expand their native ranges. These species have to adapt to low temperature conditions to avoid death. *Cinara tujafilina* (del Guercio, 1909) (Hemiptera, Aphididae) is a thermophilic anholocyclic species visible in the warm regions of Asia [12]. In its natural range this species infests above-ground segments of plants belonging to Cupressaceae throughout the year [13]. On areas where the populations of *C. tujafilina* had expanded, it demonstrated its adaptation for surviving colder winters [14,15]. This species adapted to overwintering by changing its settlement place to the root, which was first described in Polish populations [16]. Previous papers described the behavioral (migrations up/down the host plant), morphological (change in color and size of morphs) and developmental adaptations (nymphs’ adaptation to low temperatures, ability to develop up to five generations in winter), all of which ensure that this anholocyclic species is capable of surviving winter [16]. Data regarding the overwintering strategies of anholocyclic species are scarce and the question to which biochemical mechanisms allow insects to survive the uncomfortable low temperatures, is of great interest. The aim of the study was to determine (1) comparison of the chemical composition of the body of *C. tujafilina* in summer and in winter, (2) determine which putative cryoprotectants enable aphids to overwinter.

## 2. Results

### 2.1. Chemical Compositions

In this study, the differences in the chemical compositions between summer and winter types of *C. tujafilina* were determined using Fourier transform infrared (FTIR) spectroscopy. Moreover, in the winter types of *C. tujafilina*, an evolution of chemical changes during the following three months (October, November, December), was also carried out. In all obtained FTIR spectra, the same peaks corresponding to functional groups building body chemical compositions of *C. tujafilina*, were analyzed.

**Figure 1:**
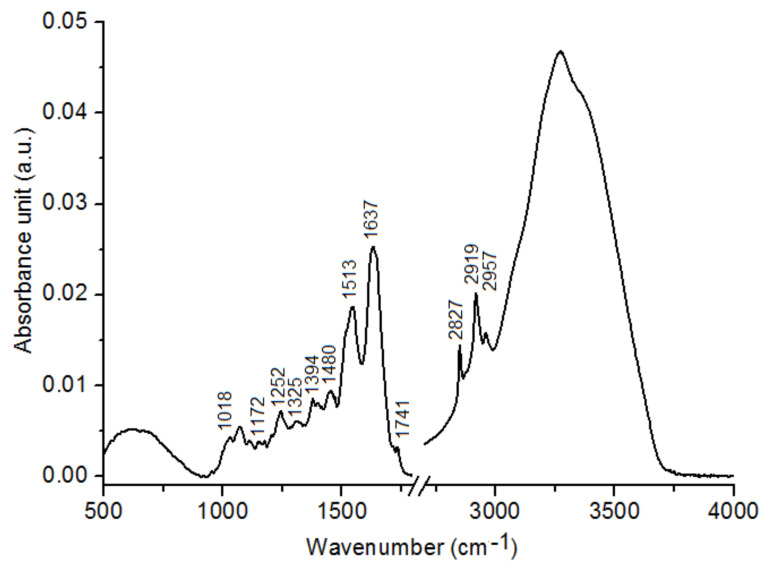
Fourier transform infrared (FTIR) spectrum of *C. tujafilina* with marked analyzed functional groups.

Figure 1 shows FTIR spectrum of *C. tujafilina* with marked peaks corresponding to: C-O stretching vibrations of glucose (1018 cm^−1^), C-O and C-C stretching vibrations or C-O-H, C-O-C deformation of carbohydrates (1172 cm^−1^), CH3 deformation vibrations of lipids (1394 cm^−1^), OH and ester carbonyl groups = C = O (1480 cm^−1^, 1252 cm^−1^, 1325 cm^−1^), amide II (1513 cm^−1^), amide I (1637 cm^−1^), C = O stretching symmetric vibrations from fatty acids and lipids (1741 cm^−1^), CH2 and CH3 vibrations from lipids (2827 cm^−1^, 2919 cm^−1^ and 2957 cm^−1^).

**Figure 2:**
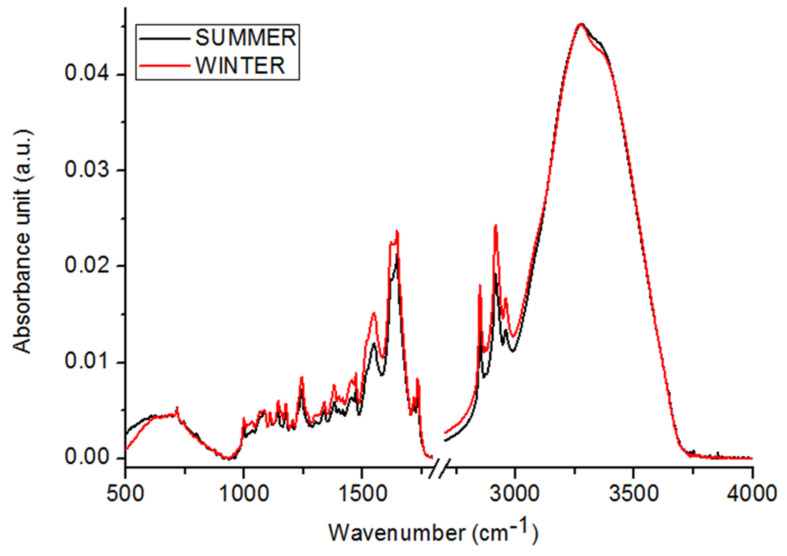
FTIR spectra of summer (black spectrum) and winter (red spectrum) of *C. tujafilina.*

Figure 2 shows FTIR spectra of winter and summer types of *C. tujafilina*. In the compared spectra, the differences in the values of maximum absorbance and in the position of the peaks, can be observed. The first, means the changes in the amount of each chemical composition, which are built by the (marked in Figure 1) functional groups. Changes in the shape of spectra and peaks’ positions, can be analyzed as a structural difference between two types of *C. tujafilina*. As we can see, in the FTIR spectrum of winter type of *C. tujafilina*, higher values of peaks originating from the carbohydrates, proteins and lipids, were observed in comparison with FTIR spectrum of summer type of *C. tujafilina*. Moreover, the most visible increases of maximum absorbance, can be seen in the IR region corresponding to proteins and lipids. The values of analyzed peaks in Table 1, were described and, furthermore, changes in the shape of spectra and the shift of peaks’ origination from the proteins and carbohydrates, were noticed between winter and summer types of *C. tujafilina*.

**Figure 3:**
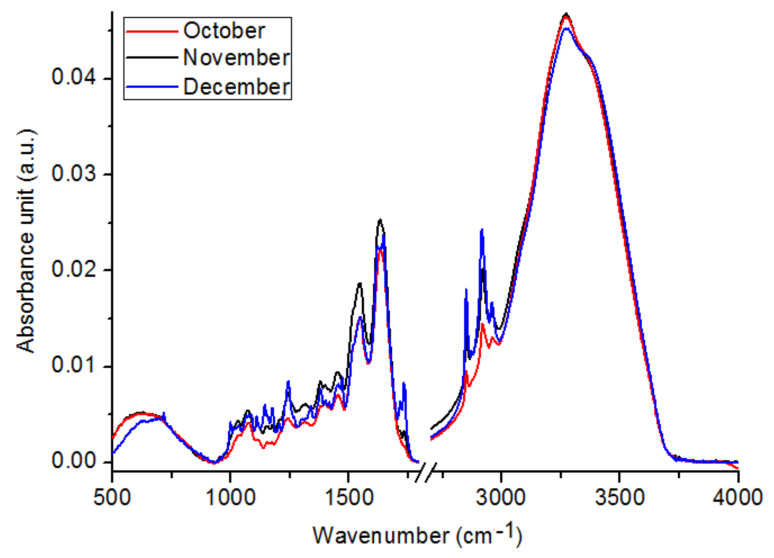
FTIR spectra of October (red spectrum), November (black spectrum) and December (blue spectrum) of *C. tujafilina*.

When we compared the amount of carbohydrates, proteins and lipids in the *C. tujafilina* collected from different winter months, we observed that the lowest level of function groups building these substances was in the FTIR spectrum of *C. tujafilina* collected in October (Figure 3). Furthermore, the *C. tujafilina* collected in November had the highest level of OH and ester carbonyl groups = C = O, amide II and amide I vibrations, while the FTIR spectrum of aphids collected in December are characterized by the highest values of maximum absorbance corresponding to carbohydrates and lipids. The values of absorbance for the peaks analyzed for October, November and December types of *C. tujafilina* in Table 2, were described.

**Figure 4:**
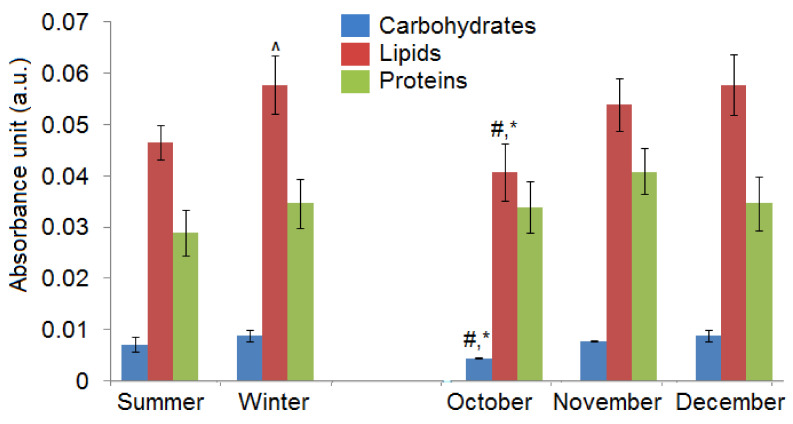
Sum of maximum absorbance of functional groups building carbohydrates (blue), lipids (red) and proteins (green). The differences are statistically significant between summer and winter (^) and between October and November (^#^), October and December (^*^), (*p* < 0.05, Tukey’s test).

Moreover, to show global changes in carbohydrates, lipids and proteins, the sum of absorbance values of functional groups building these compounds, is presented in Figure 4.

As we can see, there were only significant differences in the global amount of lipids, when we compared *C. tujafilina* collected in summer and winter. Moreover, a higher amount of all analyzed chemical compounds, in winter were observed. Furthermore, when we compared the winter months, we noticed that the smallest amount of carbohydrates, proteins and lipids were in October. In November the highest amount of proteins was visible, while in December the level of lipids is the highest.

**Figure 5:**
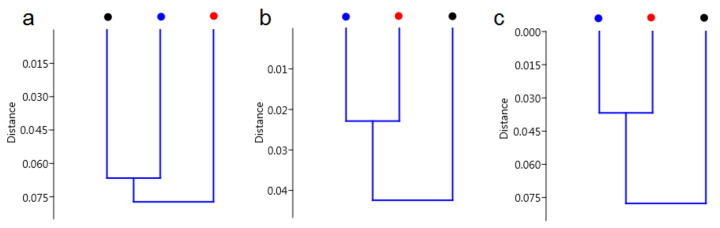
Hierarchical Clustering Analysis (HCA) analysis for selected IR region (**a**) (800 cm^−1^–1800 cm^−1^—fingerprint), (**b**) (1000 cm^−1^–1300 cm^−1^—carbohydrates) and (**c**) (2700 cm^−1^–3000 cm^−1^—lipids) for October (red dot), November (black dot) and December (blue dot) *C. tujafilina*.

**Figure 6:**
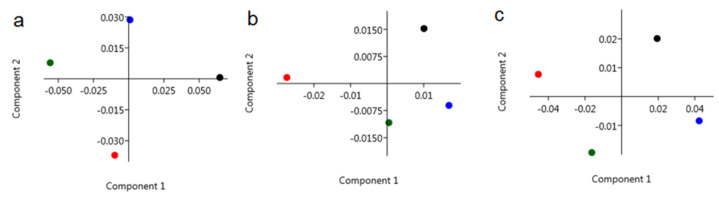
Principal Component Analysis (PCA) analysis for selected IR region (**a**) (800 cm^−1^–1800 cm^−1^—fingerprint), (**b**) (1000 cm^−1^–1300 cm^−1^—carbohydrates) and (**c**) (2700 cm^−1^–3000 cm^−1^—lipids) of summer (green dot) and winter months: October (red dot), November (black dot), December (blue dot) and of *C. tujafilina*.

To determine the similarity between *C. tujafilina* collected in October, November and December, an Hierarchical Clustering Analysis HCA analysis was performed (Figure 5). Furthermore, to obtain information about statistically significant chemical changes observed in the FTIR spectra, as well as to determine whether it is possible to distinguish the *C. tujafilina* using the chemical composition of these insects, a Principal Component Analysis PCA analysis was made (Figure 6). These analyses were made for three different IR regions: (800 cm^−1^–1800 cm^−1^—fingerprint), (1000 cm^−1^–1300 cm^−1^—carbohydrates) and (2700 cm^−1^–3000 cm^−1^—lipids), to observe the changes in the amount of spare substances in the aphid’s body, to enable it to live in low temperatures. The HCA analysis showed, that when we analyzed the “fingerprint” region of *C. tujafilina* (Figure 5a), the samples collected in November and December were similar to each other. However, taking into account the IR region corresponding to carbohydrates and lipids vibrations (Figure 5b,c), we observed similarity between samples collected in October and December.

PCA analysis of FTIR spectra of *C. tujafilina* presented in Figure 6 shows that the observed chemical changes in the *C. tujafilina* from the three months and summer, were statistically significant. Moreover, these changes made it possible to distinguish the periods in which the aphids were collected. This could mean that the evolution of the chemical composition of *C. tujafilina* is huge and that the aphids are consciously preparing for the winter. Furthermore, it is possible to distinguish the “winter types” of *C. tujafilina* when taking into account the changes in carbohydrates and lipids, as well as all “fingerprint” range characteristics for *C. tujafilina*. PCA analysis of the FTIR spectra of *C. tujafilina* collected from summer and the three winter months, showed that in the case of the fingerprint region (Figure 6a) the main chemical compositions had huge differences for summer and winter (December) for the *C. tujafilina* samples, as well as for the October and November winter months. Also, for the same pair of samples, the amount and structure of carbohydrates showed smaller similarities to each other (Figure 6b). Furthermore, the most variable in terms of the season IR regions are those corresponding to lipids (Figure 6c), where PCA analysis showed that it is possible to distinguish the FTIR spectra of *C. tujafilina* collected in summer, October, November and December.

**Figure 7:**
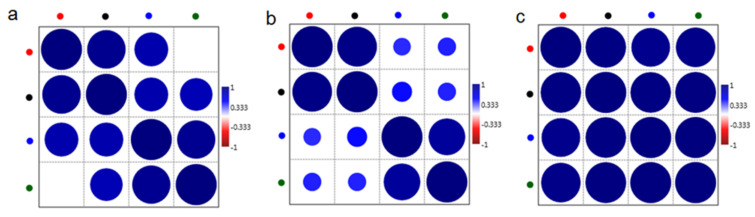
Pearson correlation test for selected IR region (**a**) (800 cm^−1^–1800 cm^−1^—fingerprint), (**b**) (1000 cm^−1^–1300 cm^−1^—carbohydrates) and (**c**) (2700 cm^−1^–3000 cm^−1^—lipids) of summer (green dot) and winter months: October (red dot), November (black dot), December (blue dot) and of *C. tujafilina*.

The Pearson correlation test (Figure 7) showed in all analyzed IR regions, a positive and significant correlation between the months in which *C. tujafilina* were collected. Only in the case of the fingerprint region (Figure 7a), correlation between summer and October samples was not visible. Moreover, the strongest correlation was noticed between the months in which *C. tujafilina* were collected and amount of lipids (Figure 7c). In the case of the carbohydrates IR region, higher correlation was visible for October, November, December and summer, than for October/summer, October/December, November/summer and November/December (Figure 7b).

### 2.2. Sugar and Polyols Analysis

Based on the HPLC analysis, we demonstrated the putative cryoprotectants in the aphid body. Glucose, trehalose, mannitol, fructose, myo-inositol and glycerol were identified in the aphid body, both in summer and winter (Figure S1) but there were differences between the samples in the amount of substances. The differences between the samples depended on the season (summer-winter) and the type of sugars and polyols. The main effect of season on chemical composition of the aphid body was significant (two-way ANOVA F_(1,24)_ = 23.73, *P* < 0.001). Moreover, the type of compound had a significant effect on the amount of substance (two-way ANOVA F_(5,24)_ = 57.29, *P* < 0.001). There was also significant interaction between season and type of compound to amount of substance (two-way ANOVA F_(5,24)_ = 7.27, *P* < 0.001).

**Figure 8:**
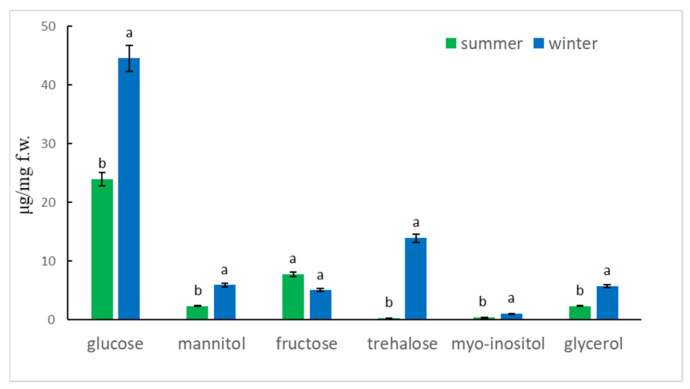
Mean cryoprotectants concentration in the body of *C. tujafilina* during summer and winter season. Values, marked with different letters, differ significantly at *p* < 0.05 (*t*-test).

Glucose had a higher amount in winter samples and significantly differed from summer. Trehalose significantly increased in the winter aphid body, while the amount of this compound in the summer samples was only trace amounts. Fructose was present in the aphid body during summer and winter but the amount was higher in summer samples. The evidence showed that the average level of mannitol increased in the aphid body during winter and was statistically different than in summer. Myo-inositol and glycerol showed significant changes, because their visible increase were noted in the winter samples (Figure 8).

PCA analysis showed the separation of summer and winter samples. The correlation of composition of sugar and polyols with the PC1 axis showed that glucose (Pearson correlation, *P* < 0.05), mannitol (*P* < 0.05), trehalose (*P* < 0.001) and glycerol (*P* < 0.05) had a major impact on the differentiation of samples and played the main role as cryoprotectants in the aphid body (Figure S2). Fructose had a significant influence on the summer samples (*P* < 0.05).

## 3. Discussion

Many insects have strategies that allow them to pass through or avoid, the adverse effects of cold weather in winter. These can be either behavioral strategies such as migration or physiological strategies such as freeze avoidance and freeze tolerance [17]. Insects prepare in advance in order to survive the winter. Most aphids lay eggs in the autumn which overwinter but the anholocyclic species of aphids must overwinter as living specimens and this has forced them to adopt other life strategies. Our research has shown that the anholocyclic species of *C. tujafilina* adapts to the cold winter conditions not only behaviorally but also physiologically. Our previous research has shown that this species changes its place of settlement on the plant, from the green parts to the root, where individuals migrate [16]. In these studies, we found that *C. tujafiline* modifies its body chemical composition in the months leading up to winter and also accumulates putative cryoprotectants. We observed a change in the chemical composition of the body between summer and winter (Figure 2). The aphid body during winter contained a higher volume of carbohydrates, proteins and lipids, in comparison with the FTIR spectrum of the summer sample of aphids. Interestingly, we also observed changes in the shape of spectra and shift of peaks’ origination from the proteins and carbohydrates, between winter and summer types; all of which meant structural changes in these substances. Seasonal changes in the chemical structure of the insect’s body showed fluctuations during the months of October, November and December (Figure 3). We also recorded seasonal changes in chemical composition, as the amount of protein, carbohydrates and lipids was lowest in October and highest in December (Figure 3). Thanks to FTIR, we were able to show changes in the structure and composition of the aphid body, which would not be possible when analyzing the total amount of sugars, proteins and lipids (Figure 4). The seasonal acquisition of high concentrations of cryoprotectants has been well characterized in many insect species especially beetles. In this case, it was found that the concentration of cryoprotectants is subject to certain characteristic fluctuation patterns [7]. The seasonal pattern of accumulation/depletion of the polyol mixture in the beetles was unusually complex. Seasonal changes in the amount of cryoprotectants were also observed in *Brevicoryne brassicaceae*, where the content of substances in the body of the aphid increased in the following months from October and reached the highest values in December and January [18]. Our research shows, however, that physiological changes are more complex and also apply to substances visible in fingerprint, not only carbohydrates and lipids. The differences in the chemical composition of the samples collected in the following months showed us how *C. tujafilina* adapts to wintering (Figure 5). Aphids feeding on the green parts of the host plant in summer, in autumn and early winter, significantly changed the chemical composition of their body (Figure 6 and Figure 7). Our previous research found that aphids were able to overwinter on the plant’s roots and survive the unfavorable low temperatures [16]. The analysis of the content of sugars and polyols showed that the body of *C. tujafilina* accumulates these substances. The accumulation of low molecular sugars and polyols is one of the major physiological mechanisms to increase cold tolerance of insects but little is known about this process in aphids. We found changes in the content of 7 compounds: glucose, mannitol, trehalose, myo-inositol, glycerol and fructose (Figure S1 and Figure 8). All substances showed an increased amount in the *C. tujafilina* body in winter, only fructose decreased. Glycerol is the most often reported polyol in literature [4], for example in beetles or moth [7,17,19] but for aphids we do not have any information, at this time. The populations of *Ips typographus* mainly accumulated glycerol and glucose [20] but Czech populations of these insects have a multiple component system of sugars and polyols consisting of 11 various compounds [7]. In *C. tujafilina*, the amount of accumulated glycerol was not very high. Probably, as in other insects, it could play an important role in the freeze avoidance strategy of these insects by permitting suppression of supercooling point to prevent body freezing [4]. Glucose, mannitol and trehalose were also the main cryoprotectants observed in *Schizaphis graminum* during the process of cold acclimation [21]. Accumulation of glucose and trehalose can play an important role in the process of stabilizing proteins and membrane lipids [21]. Glucose is the main cryoprotectant also in other insects, for example overwintering pupae of *Hyphantria cunea* [22]. Also trehalose, which is the major hemolymph sugar of insects, was noted as a key cryoprotectant in many overwintering insects such as larvae of *Cydia pomonella* [23,24]. Mannitol was detected in a trace amount in overwintering adults of *S. graminum* [21] but in *B. brassicae* it was one of the main cryoprotectants [18]. *B. brassicae* also contained myo-inositol but in a rather low amount. Myo-inositol is accumulated during diapause and overwintering in many beetles, flies and in hemolymph in planthoppers, where it could play a role in flight energy and also thermo- or, cryoprotectant for changes in physiological conditions [25,26,27,28,29]. Mannitol was also noted with trehalose and glycerol in adults of *I. typographus* [7]. Earlier studies indicated that mannitol plays a role in thermo-osmoprotection as a response to thermal stress because it accumulates in the body of insects under the influence of high temperatures [30]. It is very interesting to show the changes in the amount of fructose, which dominated in the summer trials and clearly reached the lowest level in the winter trials. This sugar was also noted in *I. typographus* where it was undetectable or reached a very low level [7]. Although the fructose level dropped significantly in the aphid body in winter, its content was relatively high in contrast to *I. typographus*. Due to fructose being one of the main constituents of honeydew in aphids [31], it suggests that overwintering aphids produce less honeydew in winter, which had already been observed in our earlier studies [16]. Previous studies showed that aphids and whiteflies used fructose as the substrate for polyol synthesis, for aphids especially mannitol [30]. Metabolic paths for fructose have to be limited, because high levels of fructose would be toxic if large quantities of this reducing sugar were absorbed by insect cells [32]. The main mechanism to decrease its amount is the conversion of fructose to polyols. Presumably, under conditions where polyols do not accumulate, fructose within the insects’ bodies is either converted to glucose or excreted in honeydew. Mannitol are always trace components in the honeydew secreted by aphids [30]. Changes in the identified components of the aphid body suggest that they could play an important role in cold acclimation of *C. tujafilina* (Figure S2).

Synthesis of cryoprotectants is supported by the accumulation of glycogen in an insect’s body during autumn feeding and also increased enzyme activity that are necessary to produce polyols. This process involves a massive conversion of glycogen to polyols and sugars and was observed in larvae of the goldenrod gall moth [17,19,33]. Aphids, as phloem-feeding insects, cannot use the wet phloem avoidance strategy during wintering, as some beetles do [7,34]. Previous research has shown that cryoprotectants may provide protection against low temperatures, even in small amounts. This is due to their ability to preserve native conformation of proteins at low temperatures [35]. Trehalose can also actively reparation the structure of proteins [36,37] and can interact with chaperones to help re-fold partially denatured proteins after thermal stress [38]. Some cryoprotectants, such as trehalose and mannitol, could serve also as potent free radical scavengers, thus protecting cells from oxidative damage [39]. The six carbon polyols do not readily cross membranes, which is one of the major reasons for their value as osmoprotectants in insect systems [40].

The relationship between the accumulation of cryoprotectants and cold tolerance was demonstrated for some Hemiptera species, for example, *Microvelia reticulata* or *B. brassicae* [18,41]. The comparative analysis of overwintering physiology of nine species of semi-aquatic bugs showed, however, that some species can reach a high level of cold tolerance without accumulating polyols for example, *Gerris odontogaster* [41]. Some of the semi-aquatic bugs use other physiological mechanism to avoid freezing, such as low hydration of the body or thermal hysteresis between the melting and equilibrium freezing points [41]. Some semi-aquatic bugs, such as *Hydrometra stagnorum*, can achieve a high level of cold tolerance by high body fluid osmolality [41]. Our study therefore cannot conclusively confirm that summer type of *C. tujafilina* are not cold tolerant and further studies should resolve this. Our research, however, indicates that winter type of *C. tujafilina* accumulates sugars and polyols as putative cryoprotectants.

Our research is the first to show, using *C. tujafilina* as an example, how aphid species that expand their ranges adapt to unfavorable low temperatures, in order to overwinter as active stages. We have shown that the chemical composition of the aphid body changes as it adapts to wintering. These changes concerned the amount of compounds and the structure of functional groups. In the FTIR spectrum of winter type of *C. tujafilina*, higher values of peaks originating from the carbohydrates, proteins and lipids, were observed in comparison with FTIR spectrum of summer type of aphids. To prevent freezing, *C. tujafilina* showed a complex sugar-polyol cryoprotectant system.

## 4. Materials and Methods

### 4.1. Aphids and Sample Collection

The aphid *Cinara tujafilina* were reared in a prepared habitat on *Thuja orientalis* at the University of Rzeszow (Poland) in natural conditions. Aphids for analyses were collected once a month and we used only wingless adults. Summer samples were taken in June from the leaves of the host plant. Winter samples of *C. tujafilina* were collected from the main stem near the root collar or from the roots of the plant. For determination of changes in chemical compositions of the aphid body, samples were collected in June (summer sample) and December (winter sample) and monthly from October to December. For biochemical analysis of polyols and sugars, summer and winter samples (each 30 individuals) were weighed. Air temperature data obtained from Tutiempo.com for Rzeszów in 2019. The average air temperature was 11.3 °C in October. It decreased to 6.4 °C in November and to the lowest value of 3.3 °C in December. The absolute minimum air temperature, the very lowest recorded temperature in each month, was −4.3, −7.4 and −4.6 °C in October, November and December, respectively.

### 4.2. Determination of Chemical Compositions

Individual aphids were placed in separate test tubes. Each sample was rubbed on a glass slide and the aphid’s body mass placed on the ATR crystal. In order to establish the differences in the chemical compositions between summer and winter types of *C. tujafilina* ATR-FTIR spectra obtained from summer and winter were compared [42,43,44,45,46]. Also the samples collected in subsequent months were compared to establish the chemical changes during three months. For each time interval (summer, winter, October, November, December) measurements were taken on 10 samples. Spectra of aphid samples were measured using a Nicolet iN10 MX microspectrometer, which has a deuterated triglycine sulphate (DTGS) detector and KBr beam splitter (Thermo Fisher Scientific, Waltham, MA, USA) with Nicolet iZ10 module, coupled with a Smart Orbit ATR one-bounce diamond crystal (diamond 30.000–200 cm^−1^) accessory. Measurements were controlled using OMNIC software (Thermo Fisher Scientific). Spectra for both background and aphid sample measurements were recorded at a resolution of 4 cm^−1^ and 64 scans in the range of 500–4000 cm^−1^. For each spectrum, baseline correction and vector normalization were made using OPUS 7.0 software (Bruker Optik GmbH, Ettlingen, Germany).

### 4.3. Determination of Sugars and Polyols

To detect the composition of sugars and polyols in the aphid body, the aphids were stored at −25 °C in two separate groups: summer samples and winter samples. Each sample (30 individuals) was homogenized in 1.5 mL of 80% ethanol and centrifuged at 12,000 *g* for 15 min. Three replicates for each sample were performed [18]. The supernatant was collected and the extract evaporated in a vacuum drying oven. To each sample, 150 μL H_2_O (HPLC) was added and the samples were cleaned by a cellulose acetate filter syringe. The sugars content and polyols content, were measured by HPLC method with refractive index detection. The chromatographic equipment SYKAM (Sykam GMBM, Eresing, Germany) consisting of sample injector S5250, pump system S1125, column oven S4120 and RI detector S3590 was used. Separation was carried out using Sugar-D column (5 µm, 4.6 mm × 250 mm; Cosmosil, Nicalai Tesque, Kioto, Japan). The separation was achieved with a mobile phase of 70% acetonitrile (ACN) in water in isocratic mode (HPLC grade, >99.9%, Chromasolv, Honeywell Riedel-de-Haën, Seelze, Germany). The flow rate was 1 mL/min at column temperature set at 30 °C. The volume of injected sample was 20 µL and 20 min was needed to complete the analysis. All determinations were performed in triplicate. The compound identification was based on the comparison of retention time with authentic standards. Quantification was performed by the external standard method. The concentration of detected sugars and polyols were presented as μg/mg fresh weight (μg/mg f.w.).

### 4.4. Statistical Analyses

In order to compare the Attenuated Total Reflectance-Fourier Transform InfraRed ATR-FTIR spectra obtained from summer and winter samples, a Principal Component Analysis (PCA) was performed, which reduced the number of data variables. The reduction was done in such a way that the data with high variance was saved. Furthermore, to obtain information about the similarity between the samples with different months, a hierarchical clustering analysis (HCA) with Euclidean distance and Ward’s algorithms was performed. These analyses were carried out for IR ranges between 800 cm^−1^ and 1800 cm^−1^ (fingerprint region), 1000 cm^−1^–1300 cm^−1^ (carbohydrates region) and 2700 cm^−1^–3000 cm^−1^ (lipids region). Moreover, to determine the correlation between fingerprint region, lipids and carbohydrates, a Pearson correlation test with *p* < 0.05, was performed. Furthermore, the differences in the amount of functional groups between measured samples, an average from all samples from each group with a standard error of the mean (±SEM) were presented. The data were analyzed by one-way ANOVA followed by the Tukey’s test. All analyses were made using PAST 3 software (developed by Oyvind Hammer, 2001). A two-way analysis of variance (ANOVA) with season (summer, winter) and chemical composition as fixed factors was made to assess the differences between the amount of sugars and polyols. The results were expressed as mean ± SE and the differences between summer and winter samples were separated by t-test. A Principal Component Analysis (PCA) was performed to determine the changes in the composition of cryoprotectants of the aphid body during summer and winter. Statistical analyses were performed using Statistica (data analysis software system), version 13 (TIBCO Software Inc., 2017, http://statistica.io.) and PAST 3 software.

## Figures and Tables

**Table 1:** Values of maximum absorbance analyzed for the summer and winter types of *C. tujafilina*. All the differences are statistically significant (*p* < 0.05, Tukey’s test).

Wavenumber (cm^−1^)	Summer Type of *C. tujafilina* (a.u.) ± SEM	Winter Type of *C. tujafilina* (a.u.) ± SEM
1018	0.00256 ± 0.00007	0.00351 ± 0.00006
1172	0.00451 ± 0.00007	0.00521 ± 0.00007
1252	0.00488 ± 0.00004	0.00613 ± 0.00003
1325	0.00407 ± 0.00010	0.00483 ± 0.00010
1394	0.00488 ± 0.00006	0.00612 ± 0.00009
1480	0.00489 ± 0.00007	0.00617 ± 0.00007
1513	0.00880 ± 0.00003	0.01189 ± 0.00008
1637	0.01975 ± 0.00010	0.02231 ± 0.00005
1741	0.00338 ± 0.00005	0.00400 ± 0.00006
2827	0.00548 ± 0.00003	0.00692 ± 0.00005
2919	0.01869 ± 0.00006	0.02327 ± 0.00010
2957	0.01342 ± 0.00005	0.01671 ± 0.00003

**Table 2:** Values of maximum absorbance analyzed for the October, November and December types of *C. tujafilina*. The differences are statistically significant between the October and November (^#^), October and December (^*^), November and December (^&^) (*p* < 0.05, Tukey’s test).

Wavenumber (cm^−1^)	October (a.u.) ±SEM	November (a.u.) ±SEM	December (a.u.) ±SEM
1018 ^#,*,&^	0.00230 ± 0.00004	0.00390 ± 0.00009	0.00351 ± 0.00006
1172 ^#,*,&^	0.00212 ± 0.00007	0.00379 ± 0.00006	0.00521 ± 0.00007
1252 ^#,*,&^	0.00428 ± 0.00006	0.00645 ± 0.00007	0.00613 ± 0.00003
1325 ^#,*,&^	0.00408 ± 0.00009	0.00590 ± 0.00007	0.00483 ± 0.00010
1394 ^#,&^	0.00606 ± 0.00010	0.00798 ± 0.00004	0.00612 ± 0.00009
1480 ^#,*,&^	0.00553 ± 0.00005	0.00769 ± 0.00010	0.00617 ± 0.00007
1513 ^#,*,&^	0.01162 ± 0.00003	0.01549 ± 0.00006	0.01189 ± 0.00008
1637 ^#,*,&^	0.02186 ± 0.00008	0.02484 ± 0.00008	0.02231 ± 0.00005
1741 ^#,*,&^	0.00123 ± 0.00010	0.00208 ± 0.00009	0.00400 ± 0.00006
2827 ^#,*,&^	0.00556 ± 0.00009	0.00751 ± 0.00010	0.00692 ± 0.00005
2919 ^#,*,&^	0.01447 ± 0.00007	0.01990 ± 0.00002	0.02327 ± 0.00010
2957 ^#,*,&^	0.01296 ± 0.00006	0.01577 ± 0.00003	0.01671 ± 0.00003

## Data Availability

Not applicable.

## Supplementary Materials

The following are available online at https://www.mdpi.com/1422-0067/22/2/511/s1, Figure S1. Profiles of sugars and polyols detected by HPLC methods. A) set of standards, B) sample of *Cinara tujafilina* extract. The peaks: 1-glycerol, 2-fructose, 3-glucose, 4-mannitol, 5-trehalose, 6-myo-inositol. Figure S2. PCA analysis of the composition of sugar and polyols of *C. tujafilina*, depending on the season (summer, winter).
