# Supplementary material for: Changes in Chemical Composition and Accumulation of Cryoprotectants as the Adaptation of Anholocyclic Aphid Cinara tujafilina to Overwintering
Source: Int J Mol Sci. 2021 Jan 6;22(2):511. doi: 10.3390/ijms22020511 (PMC7825631; doi:10.3390/ijms22020511)
Supplement: Supplementary file 1 [file ijms-22-00511-s001.pdf]

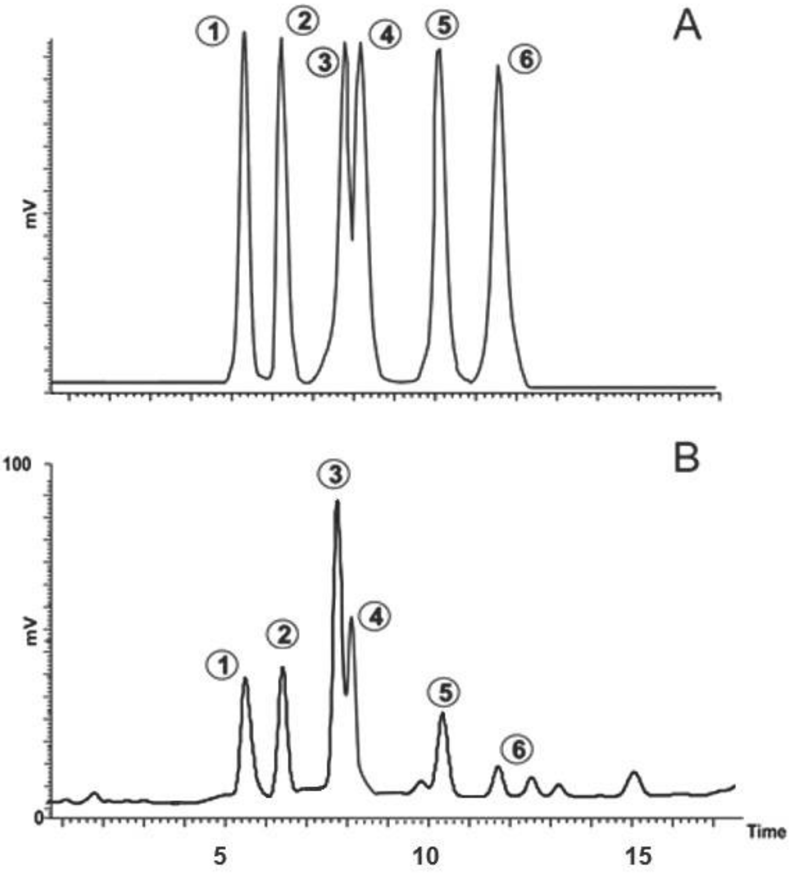

Figure S1. Profiles of sugars and polyols detected by HPLC methods. A) set of standards, B) sample of *Cinara tujafilina* extract. The peaks: 1-glycerol, 2-fructose, 3-glucose, 4-mannitol, 5-trehalose, 6-myo-inositol.

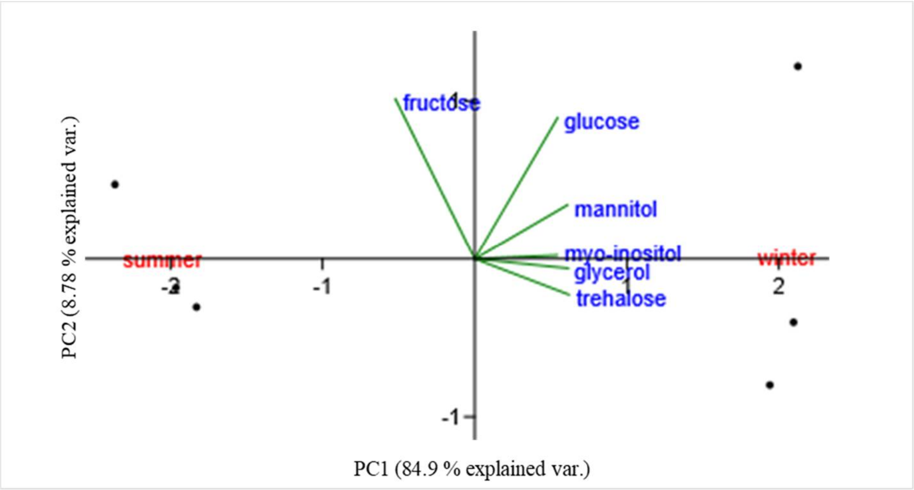

Figure S2. PCA analysis of the composition of sugar and polyols of *C. tujafilina*, depending on the season (summer, winter).
